# Preparation and Characterization of Emulsion‐Based Fucoidan‐Whey Protein Film Activated With Geranium Essential Oil for Food Applications

**DOI:** 10.1002/fsn3.71811

**Published:** 2026-04-25

**Authors:** Najmeh Oliyaei, Frahang Hameed Awlqadr, Cambyz Irajie, Somayeh Yazdanpanah, Kamyar Zomorodian, Aida Iraji

**Affiliations:** ^1^ Stem Cells Technology Research Center Shiraz University of Medical Sciences Shiraz Iran; ^2^ Department of Food Science and Quality Control, Halabja Technical College Sulaimani Polytechnic University Sulaymaniyah Iraq; ^3^ Department of Medical Biotechnology, School of Advanced Medical Sciences and Technologies Shiraz University of Medical Sciences Shiraz Iran; ^4^ Department of Medical Mycology and Parasitology, School of Medicine Shiraz University of Medical Sciences Shiraz Iran; ^5^ Department of Persian Medicine, School of Medicine, Research Center for Traditional Medicine and History of Medicine Shiraz University of Medical Sciences Shiraz Iran

**Keywords:** edible film, fucoidan, geranium essential oil, nanoemulsion, whey protein

## Abstract

Emulsion–based edible films are novel active films that can improve food quality and their shelf life due to slow release of their active ingredient. In this study, fucoidan‐ whey protein (FW) composite film with different concentration of geranium essential oil (EO) nanoemulsions [0.5% (w/w) and 1% (w/w)] were prepared (FW‐0.5 and FW‐1). The physicochemical, thermal, morphological, and mechanical properties [tensile strength (TS), elongation at break (EB)], as well as the antioxidant and antimicrobial properties, were investigated. Gas chromatography analysis detected citronellol (30.26%), geraniol (16.41%), and linalool (12.43%) as the main compounds of geranium EO and smaller amount of monoterpene hydrocarbons (such as α‐pinen, p‐cymene, and limonene) and sesquiterpene alcohols (such as globulol and pogostol). Also, the addition of a higher concentration of geranium EO nanoemulsion (1%) made the film thicker (0.13 ± 0.01 mm) than the neat FW film (0.12 ± 0.01 mm) (*p* < 0.05). The incorporation of EO nanoemulsion reduced water vapor permeability, and FW‐1 had an excellent barrier against water vapor (1.05 ± 0.28 g/m.s.Pa) when compared to the control film (1.33 ± 0.34 g/m.s.Pa) (*p* > 0.05). Moreover, active FW films loaded with EO nanoemulsion had lower TS and EB values (*p* < 0.05). TS and EB of neat FW film were 5.30 ± 0.46 MPa and 3.11% ± 0.83%, respectively, while these parameters lowered to 1.52 ± 0.65 MPa and 67.96% ± 4.70%, respectively in FW‐1 (*p* < 0.05). Active FW films had higher L* values, lower a*, b* values, and opacity in comparison with neat FW films (*p* < 0.05). Moreover, the addition of EO nanoemulsions improved thermal stability, and the active FW film (FW‐1) had higher thermal stability. SEM images exhibited a smooth surface in all FW films and a compact, dense structure in the cross‐section of FW films loaded with EO nanoemulsion. FTIR also confirmed the encapsulation of geranium EO and its compatibility with FW films. Moreover, active FW films, especially FW‐1 exhibited higher antioxidant (72.91% ± 2.53%) than FW film (35.39% ± 3.19%) and possessed stronger antimicrobial activity against Gram‐negative ( *E. coli* ), Gram‐positive ( *S. aureus* ) and fungi ( *C. albicans* ). This study suggested the potential applications of active FW film for food packaging.

## Graphical abstract

The geranium essential oil (EO) nanoemulsion was incorporated into fucoidan‐whey protein film. Active fucoidan‐whey protein film showed higher mechanical, antioxidant, and water barrier properties. Fucoidan‐whey protein film loaded with geranium EO nanoemulsion exhibited antimicrobial and antifungal activities.

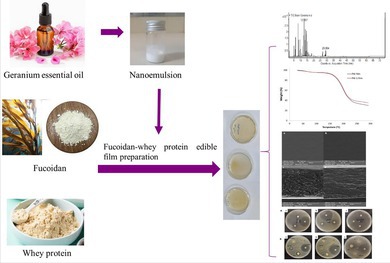

## 1 Introduction

In recent years, biopolymer‐based packaging has gained much attention due to its biodegradability and safety impact on the environment. In this regard, edible films are usually made from polysaccharides, proteins, and lipids as eco‐friendly alternatives to traditional synthetic packaging materials (Sogut et al. 2019). Edible films are thin layers of biopolymer that act as a protective layer on the food surface, inhibit microbial growth, and extend the food's shelf life. Edible films are promising carriers for active ingredients, and their antioxidant and anti‐microbial activities represent new forms of active packaging (Sayadi, Abedi, and Oliyaei 2025).

In recent years, whey protein‐based edible films have gained attention for their wide range of applications in food and can serve as carriers for antimicrobial agents. However, whey protein films have some drawbacks due to their low vapor barrier property and low mechanical resistance in comparison with synthetic polymers (Papadaki et al. 2022). Therefore, the film characteristics of whey protein should be improved to increase its competitiveness in the edible packaging market. Thus, combining with other polymers and crosslinking methos (using transglutaminase) have been applied (Jiang et al. 2020). In this regard, production of composit whey protein films with polysaccharides are promising technique to over these limitations. The globular structure of whey protein possesses hydrophobic groups, such as thiol, which can facilitate the interaction with polysaccharides during heat‐induced gelation. Therefore, improve their barrier and mechanical attributes. Thus, the development of whey protein edible films with usual polysaccharides offers a novel approach (Sogut 2020). For instance, whey protein‐starch based active film loaded with lachnanthocarpone (Ramirez‐Álvarez et al. 2025), whey protein‐pectin (Ke et al. 2025), whey protein‐gum Arabic film (Lu et al. 2025), and whey protein‐balangu gum film (Mohammad Amini 2025).

Among various resources, algal polysaccharides are renewable, abundant, and biodegradable which are found in seaweeds up to 70%. Seaweed polysaccharides have physical structure properties in brown macroalgae and have a broad spectrum of biological activities (Oliyaei et al. 2025). Fucoidan is a fucose‐rich sulfated polysaccharide found in brown seaweeds and is composed of fucose, galactose, glucose, mannose, xylose, rhamnose, and uronic acids. The structural composition of fucoidan varies among different algal species, seasons, and harvesting conditions (Oliyaei et al. 2023). This water‐soluble polysaccharide is Food and Drug Administration (FDA) approved and can be used in food ingredients at levels up to 250 mg/day (Oliyaei et al. 2023). Due to its wide range of potential health benefits and biological properties, such as anti‐infammatory, antioxidant and anti‐microbial effects, fucoidan has attracted significant attention for use in the food and pharmaceutical industries, in particular, the negative sulfate groups of its structure provide binding sites for interaction with other polymers (Oliyaei et al. 2023; Oliyaei et al. 2024). This novel polysaccharide can be a good candidate for the development of biocomposite films and mats with other polymers, to improve its film forming (Gomaa, Hifney, et al. 2018; Hifney et al. 2018).

In addition, essential oils (EO) are antioxidant and antimicrobial compounds that have been incorporated into films to improve food quality and prolong the shelf life by slowing their release across the film (Sayadi, Abedi, and Oliyaei 2025). Moreover, direct utilization of EOs is often limited due to their strong flavor which has an impact on consumer acceptance. To avoid these problems and cover their odor, EO is incorporated into the film matrix (Behbahani et al. 2020). However, the method has some challenges due to its inherent hydrophobicity, volatility, low solubility, and low efficiency. Therefore, the encapsulation of EO is a promising solution that offers higher stability and efficacy (Abedi et al. 2024). Emulsions are suitable systems for encapsulating EOs into edible films or coatings, providing optimal performance due to the slow release of EOs (Abedi et al. 2024). Oil in water (O/W) nanoemulsions consist of small oil droplets dispersed within an aqueous continuous phase and are promising tools for improving food preservation through edible films or coatings, creating a protective layer on food surfaces (Ul Islam et al. 2024). Additionally, the dispersion of oils in O/W emulsion can enhance the film's resistance to water and act as a carrier for water‐insoluble active ingredients, protecting them and improving their dispersion in the film matrix (Sun et al. 2025). Also, the nanodroplet size of nanoemulsions creates higher performance due to the higher surface area. Therefore, emulsion‐based edible films have gained much attractive attention (Abedi et al. 2024).

Geranium EO is popular due to its many therapeutic properties and has numerous applications in the food industry. Phenolics, such as phenols and flavonoids, are the largest phytochemical compounds in geranium EO, responsible for its antimicrobial activity (Wang et al. 2022). Geranium EO is obtained from aromatic and medicinal Geraniaceae family plants with a wide application in perfumery, cosmetics, aromatherapy, pharmaceuticals and food industries. Numerous investigations confirmed the biological features including antifungal, antibacterial, anti‐inflammatory, anti‐cancer, anti‐depressant, antioxidant, and antidiabetic properties. Moreover, the improvement of blood circulation, treats congestion, cleans the lymphatic system, strengthens the immune system, and is effective in combating nervousness, constipation, insomnia, anxiety and high blood pressure (Value Essential Oil from Geranium 2019). Recently, Ren et al. (2025) prepared alginate‐based film containing geranium EO emulsion with good thermal stability and antibacterial potential.

To the best of our knowledge, no study has been reported about the active fucoidan‐whey protein composite film loaded with geranium EO nanoemulsion. Therefore, the aim of this study was the development of fucoidan‐whey protein (FW) edible film loaded with geranium EO nanoemulsion. Thus, geranium EO was encapsulated in an O/W nanoemulsion system and then incorporated into the FW film. Then, their physicochemical (thickness and color), mechanical, thermal, morphological, antioxidant, and antimicrobial properties were investigated.

## 2 Materials and Methods

Fucoidan derived from brown seaweed *Focus versiculos* (MW 120 kDa; CAS 9072‐19‐9) was obtained from Persian Gulf Algae Development Technology Company. Whey protein (a mixture of whey protein isolate and the liquid material created as a by‐products of cheese production) was purchased from QUELAB (Canada, CAT. No. QB‐39‐5271). Glycerol and Tween 80 were purchased from Merck Co. (Germany). The geranium essential oil was purchased from the Iran Essential Oil Research Institute.

### 2.1 GC–MS Analysis of Geranium Essential Oil

The GC/MS analysis was performed using an Agilent 7000 triple quad mass spectrometer (Agilent Technologies, USA) coupled with an Agilent 7890A gas chromatography (Agilent Technologies, USA) instrument. The oven temperature was initially set at 70°C and then ramped up to 280°C, where it was held for 4 min. The injector and auxiliary temperatures were maintained at 250°C and 280°C, respectively. The division ratio was 1/60, and the carrier gas, helium, flowed at a rate of 1.2 mL/min. The operating parameters of the mass spectrometer were as follows: ionization voltage at 70 eV and a mass range of 40–650 atomic mass units (amu). Retention indices (RIs) were determined for all compounds using the retention times of n‐alkanes (C_6_–C_24_) injected after the oil under the same conditions.

### 2.2 Nanoemulsion Preparation

The geranium EO nanoemulsion was prepared according to the method described by Gholamhosseinpour, Hashemi (Gholamhosseinpour et al. 2023). Geranium EO (6% w/w), tween 80 (6% w/w EO), and water were mixed and subjected to ultrasonic emulsification using a 29 kHz sonicator (BANDELIN, SONOPULS, Germany) for 5 min.

#### 2.2.1 Droplet Size of Nanoemulsion

The droplet size of diluted geranium EO nanoemulsion (1:100 v/v) was measured by dynamic light scattering (DLS, SZ 100, Horiba, Kyoto, Japan) at ambient.

### 2.3 Preparation of Films

FW film was prepared according to the method described by Sogut (2020) with slight modifications. First, the stock solution of whey protein at 5% (w/v) was dissolved in distilled water, and the pH was adjusted to 8.0 using 2 N NaOH. The mixture was stirred at 90°C until completely dissolved. Moreover, the stock solution of fucoidan (1% w/v) was prepared by dissolving fucoidan in distilled water and stirring until completely dissolved. Then, the film‐forming solution was developed by mixing fucoidan and whey protein (1:1 v/v) and stirring for an additional 25 min, with glycerol (35% based on polymer powder) added as the plasticizer. Finally, the film solution was cast in 8 mm plates and dried for 72 h at room temperature (26°C ± 2°C). Active FW films were also produced as the same method described above within the dropwise addition of geranium EO nanoemulsion into the FW film solution. The final concentration of EO nanoemulsion was 0.5% v/v and 1% v/v and active films were FW‐0.5 (containing 0.5% geranium EO nanoemulsion) and FW‐1 (containing 1% geranium EO nanoemulsion).

### 2.4 Characterization of Edible Films

#### 2.4.1 Film Thickness

The thickness of the films was measured using a micrometer (Mitutoyo No. 293–766, Tokyo, Japan) and measurements were taken at three distinct points.

#### 2.4.2 Water Vapor Permeability (WVP)

WVP was measured according to the method described by Sayadi, Abedi, and Oliyaei (2025). Test cups (*D* = 12 mm) were filled with CaCl_2_ (2.5 g) and films were sealed over the cups. Then, the cups were placed in a desiccator containing sodium chloride saturation (75% RH) at 25°C, creating a pressure differential of 1753.55 Pa across films. WVP was calculated as Equations (1) and (2):

(1) $$\text{WVTR}=\frac{\text{Slop}}{A}$$

(2) $$\mathrm{VP}=\frac{\text{WVTR}\times L}{∆P}$$

where WVTR is determined by the determined slope (g/m^2^), *A* (m^2^) represents the film area, *L* is the film thickness (m), and Δ*P* is the partial water vapor pressure difference (Pa) between the two sides of the film.

#### 2.4.3 Mechanical Properties

The FW films tensile strength (TS) and % Elongation at break were evaluated using (TA‐XT2, Stable Microsystems, Surry, UK) according to the method described by Zhang et al. (2020). Each specimen (1 × 4.5 cm) was tested with the crosshead speed of 1 mm/min and the initial grip separation of 40 mm in duplicate. TS and EB were calculated according to the Equations (3) and (4):

(3) $$\mathrm{TS}=\frac{\text{Maximum force}\;\left(N\right)}{A\;\left(m^{2}\right)}$$

(4) $$\mathrm{EB}\;\left(\%\right)=\frac{\text{Film elongation}}{\text{Initial length}}\times 100$$

#### 2.4.4 Color Measurements and Opacity

The color of FW based films was measured using a colorimeter and lightness (L*), redness (a*) and yellowness (b*) were measured. To calculate the opacity, the films were cut into 1 × 4 cm and placed directly in glass cuvettes, and UV absorption of the films was determined at 600 nm using a spectrophotometer (Agilent Technologies, USA) and an empty glass cuvette as a reference (Abedi et al. 2024). The opacity of the film was measured by the following Equation (5):

(5) $$\text{Opacity}=\frac{A600}{X}$$

where A600 = absorbance of the film at 600 nm, *x* = thickness of the film in mm.

#### 2.4.5 Fourier Transform Infrared Spectroscopy (FTIR)

The FTIR spectra of films were measured using FTIR spectrophotometer (Tensor II, Bruker, Germany) in the wavenumber region 400–4000 cm^−1^.

#### 2.4.6 Thermogravimetric Analysis (TGA)

The thermal property of FW films was measured using (A Mettler‐Toledo instrument, TGA2; Sterzenbach, Switzerland). The measurement was running from 35°C up to 800°C at a heating rate of 10°C/min and under a nitrogen atmosphere.

#### 2.4.7 Morphology

The surface and cross‐section morphology of films were observed using scanning electron microscopy (SEM) (Tescan‐Vega 3, Czech Republic electron microscope). The film samples were cut (1 × 1 cm), mounted on an aluminum stub using double‐sided tape, and then coated with a gold layer. The samples were imaged on an accelerating voltage of 15 kV.

#### 2.4.8 Determination of the Antioxidant Capacity

The free radical‐scavenging properties of the developed edible films were quantified using the DPPH (2,2 diphenyl‐picrylhydrazyl, Aldrich, USA) free radical‐scavenging activity method according to the previously reported procedures with modification (Abedi et al. 2024). Each film (about 25 mg) was dipped in 5 mL of distilled water containing 20 μL of tween 80 for 1 h and centrifuged (4000 rpm for 5 min) to obtain the film extract. Then, film extract (0.5 mL) was blended with 1 mL of the DPPH solution (0.1 mM in methanol) and kept in the dark at room temperature for 30 min. The absorbance was measured using a UV‐vis spectroscopy at 517 nm, and methanol was used as a blank control. The scavenging activity was conducted as follows Equation (6):

(6) $$\text{DPPH radical scavenging activity}\;\left(\%\right)=1-\frac{A_{s}}{A_{c}}\times 100$$

where *A*_s_ and *A*_c_ were the measured absorbance of the sample and control, respectively.

#### 2.4.9 Determination of Antimicrobial Activities

The qualitative antimicrobial activity of films was evaluated by agar diffusion method according to the zone inhibition in agar medium as previously described (Ramos et al. 2012). Films were assessed for their antimicrobial capacity against standard strains of bacteria, including *Staphylococcus aureus* (ATCC 25923) and *Escherichia coli* (ATCC 25922), as well as the yeast species *Candida albicans* (ATCC 10261). So, films were cut into 12 mm diameter disks and sterilized under UV light for 20 min on each side. Then, film disks were placed on Muller Hinton Agar (Himedia, Mumbai, India) in petri dishes that had been seeded with 0.1 mL of inoculum containing approximately 10^5^ CFU/mL of bacterial or yeast cells. Additionally, film disks without the incorporation of EO and antimicrobial disks (ciprofloxacin, fluconazole) were tested under similar conditions as a control. The petri dishes were examined for zone of inhibition after 24 h incubation at 37°C. Afterwards, the zones of inhibition of the film disks on the plates were examined via measuring their diameter. Moreover, antimicrobial activity of the EO was assessed using a disk diffusion method. Blank disks were loaded with 10 and 20 μL of the EO and placed on inoculated agar plates. All the tests were performed in duplicate.

### 2.5 Statistical Analysis

The results were evaluated by analysis of variance (ANOVA), followed by Duncan's multiple range test (*p* < 0.05) using SAS software (SAS Institute, Cary, NC, USA). All measured values were presented as mean ± SD.

## 3 Results and Discussion

### 3.1 Geranium EO Analysis

**FIGURE 1:**
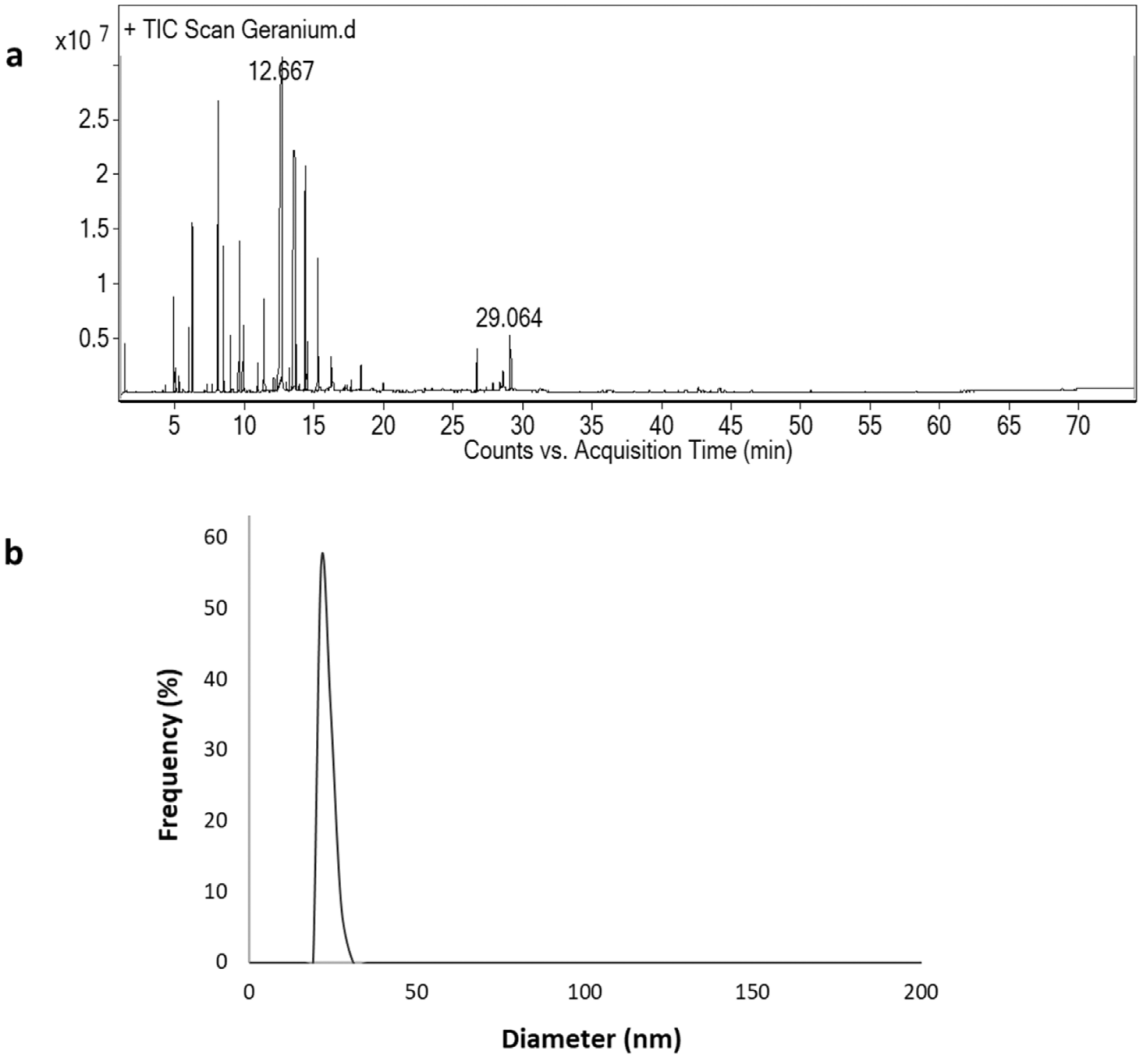
Chromatograph of geranium EO (a) and droplet size of geranium EO nanoemulsion (b).

The analysis of geranium EO was performed using GC‐MS, and the chromatogram of the EO is presented in Figure 1a and Table 1. The GC‐MS analysis of geranium EO showed that the major constituents identified include citronellol (30.26%), geraniol (16.41%), and linalool (12.43%), all of which are oxygenated monoterpenes known for their aromatic and bioactive properties that account for over 59% of the EO oil's composition. In addition, monoterpene hydrocarbons such as α‐pinene (1.52%), p‐cymene (1.14%), and limonene (3.44%) were detected in smaller amounts, contributing to the EO volatility. Sesquiterpene alcohols such as globulol (1.45%) and pogostol (1.77%) were present in minor quantities, along with various esters including citronellyl formate (8.50%) and geranyl formate (4.30%), which enhance the oil's olfactory complexity. The presence of rose oxides (cis and trans, 4.43%) further contributes to the characteristic floral scent of geranium oil.

**TABLE 1:** Chemical composition of geranium EO based on GC‐MS analysis.

No.	Compound	Amount %	Kovats index
1	α‐Pinene	1.52	975
2	p‐Cymene	1.14	1020
3	Limonene	3.44	1024
4	Linalool	12.43	1095
5	Rose oxide‐cis	3.20	1106
6	Rose oxide‐Trans	1.23	1122
7	Menthone	4.16	1148
8	Iso‐Menthone‐iso	1.61	1158
9	α‐Terpineol	0.78	1186
10	Dihydro‐citronellol	3.01	1194
11	Unknown	0.74	—
12	Citronellol	30.26	1223
13	Geraniol	16.41	1249
14	Geranial	1.26	1264
15	Citronellyl formate	8.50	1271
16	Neryl formate	1.18	1280
17	Geranyl formate	4.30	1298
18	Dihydro‐citronellol acetate	0.86	1319
19	Unknown	0.74	—
20	Globulol	1.45	1590
21	Pogostol	1.77	1651

Overall, the chemical profile suggests that geranium essential oil is rich in oxygenated monoterpenes, which are largely responsible for its pleasant fragrance and therapeutic potential, particularly as antimicrobial and antioxidant agents. Our results were in agreement with Dos Santos et al. (2024), who detected citronellol, cis‐geraniol, β‐linalool, citronellyl, and linalool in geranium EO. Medjdoub et al. (2025) similarly identified geraniol (23.5%) and citronellol (19.4%), linalool (8.3%), iso menthone (7.3%), citronellyl formate (6.7%) as the main components of *Pelargonium graveolens* EO and authors reported that *P. graveolens* EO composed of high level of oxygenated terpenoids (92.6%) including alcohol terpenoids (58.6%) and ester terpenoids (21.7%.) El‐Otmani et al. (2024) also detected geraniol (22.83%), beta‐citronellol (19.51%), naphthalenemethanol (15.36%), and geranyl tiglate (9.38%), as the main constituents of *P. graveolens* flower essential oil.

### 3.2 Size of Nanoemulsion

The dynamic light scattering (DLS) of the size distribution of the nanoemulsion, as presented in the figure, displays a single narrow peak with a diameter of approximately 15–20 nm. The presence of a single peak with a narrow distribution indicates a monodisperse system with particles of the same size, which suggests effective of the nanoemulsion (Figure 1b). Ultrasound is one of the most effective high‐energy homogenization techniques for the production of nanoemulsion that provides some advantages such as higher stability, bioavailability, and encapsulation efficacy. At high intensity ultrasonic waves, microjets and cavitation forces break the macroemulsion to nanoemulsion and increase the number of droplets (Sayadi, Abedi, Oliyaei, and Mousavifard 2025). In recent years, there has been an increase in the use of ultrasound emulsification of EOs for the development of active films. For instance, Rashid et al. (2023) produced curcumin and orange EO nanoemulsions using an ultrasound method to develop a pullulan‐based film. The droplet size of the nanoemulsion was in the range of 214.60–222.54 nm. Ashraf et al. (2025) developed pullulan/carboxymethyl chitosan active film loaded with zein‐turmeric EO nanoemulsion. The authors used ultrasound emulsification to produce nanoemulsion with a droplet size of 194.23 ± 0.41 nm. Liu et al. (Ramos et al. 2012) also generated an O/W ginger nanoemulsion using ultrasound, achieving an average droplet size of 176.4 ± 1.2 nm, to be inserted into xylan and polyvinyl alcohol film. Recently, Sayadi et al. (2024) also developed a sage seed gum film incorporated with a lemon verbena EO nanoemulsion with a droplet size of 94.3 nm. Moreover, Torres Neto et al. (2024) produced a nanomeulsion with mixture of EOs such as lemongrass and thyme at different concentrations. They observed the average droplet size of 68.88 ± 2.84 nm with 20.5 mg/mL of EO. In similar, Jesser et al. (2020) reported that the nanoemulsion of geranium EO obtained from ultarsound treatment had droplet size of 13.58 nm.

### 3.3 Film Characteristics

#### 3.3.1 Film Thickness

Film thickness is one of the most important edible film parameters that has an influence on mechanical and barrier properties of films (Almasi et al. 2020). According to Table 2, the FW film's thickness ranged from 0.12 ± 0.01 to 0.13 ± 0.01 mm and the incorporation of EO nanoemulsion into the FW matrix had no great effect on thickness (*p* > 0.05). The unchanged thickness of films after incorporation of EO nanoemulsion (at low concentration) might be related to the loss of oil phase during film preparation and formation (Almasi et al. 2020; Acevedo‐Fani et al. 2015). However, slight increase in thickness (0.13 ± 0.01 mm) was observed at higher concentrations of geranium EO nanoemulsion (FW‐1) which was related to the higher dry matter content of the matrix as a result of EO nanoemulsion (Abedi et al. 2024). Film thickness varies with the composite and additives (such as emulsifiers, plasticizers, and active agents) because high total solid content promotes film thickness (Vilas Dhumal et al. 2019). Additionally, higher viscosity of film forming solution can enhance thickness (Abedi et al. 2024). Several studies claim that adding EO or EO emulsion to the film matrix can increase film thickness (Zhou et al. 2021; Kazak and Tugrul 2024). Our results were in agreement with Wang et al. (2022) who observed that the thickness of cassava starch film was increased by increasing geranium EO concentration from 0.5% to 2%. Pratiwi et al. (2024) also reported an increase in whey protein thickness when the EO concentration was increased to 0.3%. Sajimon et al. (2023) observed a similar trend in film thickness after addition of EO. Authors reported that whey protein films incorporated with oregano EO had higher thickness.

**TABLE 2:** Thickness, water vapor permeability, and mechanical properties of fucoidan‐ whey protein films loaded with geranium EO nanoemulsion.

Films	Thickness (mm)	WVP (g/m.s.Pa)	TS (MPa)	EB (%)
FW	0.12 ± 0.01^b^	1.33 ± 0.34^a^	5.30 ± 0.46^a^	88.69 ± 5.40^a^
FW‐0.5	0.12 ± 0.01^b^	1.27 ± 0.26^a^	3.11 ± 0.83^b^	76.25 ± 7.95^ab^
FW‐1	0.13 ± 0.01^a^	1.05 ± 0.28^a^	1.52 ± 0.65^b^	67.96 ± 4.70^b^

*Note:* Data are expressed as mean ± SD. Different letters in each column indicate a significant difference between samples (*p* < 0.05). FW: Fucoidan‐whey protein film; FW‐0.5: Fucoidan‐whey protein film loaded with 0.5% geranium essential oil nanoemulsion; FW‐1: Fucoidan‐whey protein film loaded with 1% geranium essential oil nanoemulsion.

#### 3.3.2 Water Vapor Permeability (WVP)

WVP values of FW films are presented in Table 2. According to the results, the WVP value of neat FW film (control) was 1.33 ± 0.34 g/m.s which was comparable to active FW films containing EO nanoemulsion. It seems that the WVP value of FW film loaded with EO nanoemulsion was reduced slightly as EO concentration increased; however, the addition of geranium EO nanoemulsion had no remarkable effect on the WVP value (*p* > 0.05). The WVP value of FW‐0.5 and FW‐1 was 1.27 ± 0.26 and 1.05 ± 0.28 g/m.s.Pa, respectively. The WVP of film is strongly related to the interaction of water vapor with polymer, which results in diffusion and solubility coefficients dependent on the partial pressure of water vapor. Therefore, the hydrophilicity and hydrophobicity of the polymer is crucial (Gomaa, Fawzy, et al. 2018). These results suggested that EO nanoemulsion had not significant effect on the reduction of WVP value.

The WVP value of edible films is affected by the hydrophilicity and hydrophobicity of polymers, the cracks in the film structure, the thickness, and the interaction between polymers and functional ingredients (Zhang et al. 2020). The hydrophilic sites on the protein structure create interactions with water molecules. Therefore, the insertion of functional groups into the protein‐based films can change their barrier against water vapor diffusion (Mohammadi et al. 2020). Furthermore, the presence of Tween 80 in EO nanoemulsion creates polar sites for interaction with whey protein and fills free spaces leading to the reduction of WVP value. However, the incorporation of the higher amount of emulsion might have the reverse effect and increase the WVP value due to the higher polarity of tween 80 (Amjadi et al. 2021). Similarly, Gomaa, Fawzy, et al. (2018) reported that the alginate‐fucoidan film had a high WVP value, attributed to the inherent hydrophilicity of alginate and fucoidan due to their hydroxyl and carboxyl groups. Moreover, Pires et al. (2025) have observed that the protein edible film loaded with 2% oregano (Origanum compactum) EO had a lower WVP than the control film. Pratiwi et al. (2024) also reported that ginger EO concentration had an influence on whey protein‐ tapioca starch film characteristics and the addition of 0.5% ginger EO decreased WVP value.

#### 3.3.3 Mechanical Properties

The mechanical properties of films such as TS (strength of film) and EAB (flexibility of film) are critical factors that determine the structural integrity and resistance gainst deformation. TS and EAB are related to the polymer molecule, film matrix and generation condition. However, it is well known that polysaccharide‐based films have good TS, which can be equal to that of plastic films, while protein‐based films have poor mechanical attributes and brittle features (He et al. 2025). The TS and EB of FW‐based films are shown in Table 2. According to the results, the EO nanoemulsion concentration had a significant influence on the film's mechanical attributes. As the quantity of geranium EO nanoemulsion in FW film was raised, the film's TS value was found to decrease (*p* < 0.05). The TS value of FW film (without EO nanoemulsion) was 5.30 ± 0.46 MPa. While the incorporation of EO nanoemulsion significantly decreased the TS values (*p* < 0.05) and lowered 3.11 ± 0.83 MPa in FW‐0.5. The lowest TS (1.52 ± 0.65 MPa) was obtained in FW with a higher concentration of EO nanoemulsion (FW‐1) (*p* < 0.05). However, there were no significant differences between TS value of FW‐0.5 and FW‐1 (*p* > 0.05). Furthermore, it was found that the EB value was drastically reduced when the geranium EO nanoemulsion level increased (*p* < 0.05), and the active FW films had a lower EB value compared to the neat FW film. The EB value of neat FW film was 88.69% ± 5.40%, which decreased to 76.25% ± 7.95% in FW‐0.5. Also, the EB value shows a decreasing trend until the geranium EO nanoemulsion reaches 1% (67.96% ± 4.70%) (*p* < 0.05). It was observed that the addition of low concentration of geranium EO nanoemulsion had no significantly impact on EB value of FW films (*p* > 0.05). In similar, Mohammadi et al. (2020) reported the reduction of TS of whey protein film loaded with cinnamon EO (free and emulsion form), which was attributed to the plasticizer of emulsion that can easily diffuse into polymer chains, lower the brittleness of film and form a strong structure instead of weak network of polymer‐oil interaction. Moreover, EOs can interfere with polymer chain‐ chain interactions and make the more flexible structure with lower TS (Jamróz et al. 2018). It should be considered that edible films have lower mechanical resistance that plastic, however, our results were in agreement with other edible films mechanical properties. Ghadetaj et al. (2018) who claimed that whey protein films loaded with 1% emulsion of Grammosciadium ptrocarpum Bioss. EO had the lowest TS value (2.21 MPa) due to the inter‐molecular interaction of EO with protein chains. Amjadi et al. (2021) also reported the decreasing trend in TS value of whey protein films by addition of emulsion of *Citrus sinensis* peel EO and the TS values were varied between 2.35 and 0.81 MPa based on the concentration (2.5% and 5%) emulsion and nanoemulsionforms of EO.

#### 3.3.4 Color Parameters and Opacity

Color parameters and opacity of FW‐based edible films are shown in Table 3. According to the results, the lightness (L*) factor of all FW films was relatively high and the incorporation of geranium EO nanoemulsion enhanced the lightness of FW films significantly (*p* < 0.05). The L* value of the control film was 61.60 ± 2.67, while it was 64.70 ± 3.06 and 65.30 ± 2.63 in FW‐0.5 and FW‐1, respectively (*p* < 0.05). However, there were no significant differences between the lightness of FW‐0.5 and FW‐1 (*p* > 0.05). The a* value (redness) of FW was higher (0.30 ± 1.16) than active FW films loaded with geranium EO nanoemulsion (*p* > 0.05). The a* values of FW‐0.5 and FW‐1 were −0.50 ± 0.85 and −0.20 ± 1.14, respectively. Additionally, the addition of geranium EO nanoemulsion greatly reduced the yellowness (b*) of FW‐based films (*p* < 0.05). The b* value of FW (control) was 19.30 ± 2.42, while it was lowered to 13.80 ± 3.79 and 14.50 ± 1.84 in FW‐0.5 and FW‐1, respectively. Moreover, higher concentrations of EO nanoemulsion had no significant effect on color parameters (*p* > 0.05). Results of opacity also revealed that the incorporation of geranium EO nanoemulsion had no remarkable influence on opacity, however, the opacity of FW films slightly reduced (*p* > 0.05). The opacity of FW (control) was 4.90 ± 0.09, while it was 4.61 ± 0.53 and 4.53 ± 0.32 in FW‐0.5 and FW‐1, respectively. Packaging materials should prevent the deterioration of food quality from light and can block UV rays. Protein films containing a high quantity of aromatic amino acids exhibiting which are capable of absorbing UV radiation and exhibit a good barrier to UV. Additionally, the presence of plasticizers in film formulation effectively destroys the hydrogen bond network and creates transparent films (Pouralkhas et al. 2023). The light and yellowness of FW films were attributed to the light color of fucoidan and the yellowness of whey protein, respectively. In addition, the higher lightness, lower redness, and yellowness of active FW films was attributed to the geranium EO color which is relatively light. The color parameters of edible films are influenced by the type and concentration of biopolymers and their composition. For instance, Amjadi et al. (2021) reported that the addition of orange peel EO nanoemulsion into the whey protein decreased the lightness factor. Pouralkhas et al. (2023) reported that the interaction between fucoidan‐ gelatin creates cross‐links between polymers and produces the new structure. Our results were in agreement with Galus and Kadzińska (2016) who reported that the whey protein films loaded with rapeseed oil had higher opacity in comparison with neat whey protein film which was due to the light scattering from oil droplets dispersed in the whey protein matrix.

**TABLE 3:** Color properties and antioxidant activity of fucoidan‐whey protein films loaded with geranium EO nanoemulsion.

Films	L*	a*	b*	Opacity	Antioxidant activity (%)
FW	61.60 ± 2.67^b^	0.30 ± 1.16^a^	19.30 ± 2.42^a^	4.90 ± 0.09^a^	35.39 ± 3.19^c^
FW‐0.5	64.70 ± 3.06^a^	−0.50 ± 0.85^a^	13.80 ± 3.79^b^	4.61 ± 0.53^a^	62.68 ± 1.87^b^
FW‐1	65.30 ± 2.63^a^	−0.20 ± 1.14^a^	14.50 ± 1.84^b^	4.53 ± 0.32^a^	72.91 ± 2.53^a^

*Note:* Data are expressed as mean ± SD. Different letters in each column indicate a significant difference between samples (*p* < 0.05). FW: Fucoidan‐whey protein film; FW‐0.5: Fucoidan‐whey protein film loaded with 0.5% geranium essential oil nanoemulsion; FW‐1: Fucoidan‐whey protein film loaded with 1% geranium essential oil nanoemulsion.

#### 3.3.5 FTIR Analysis

**FIGURE 2:**
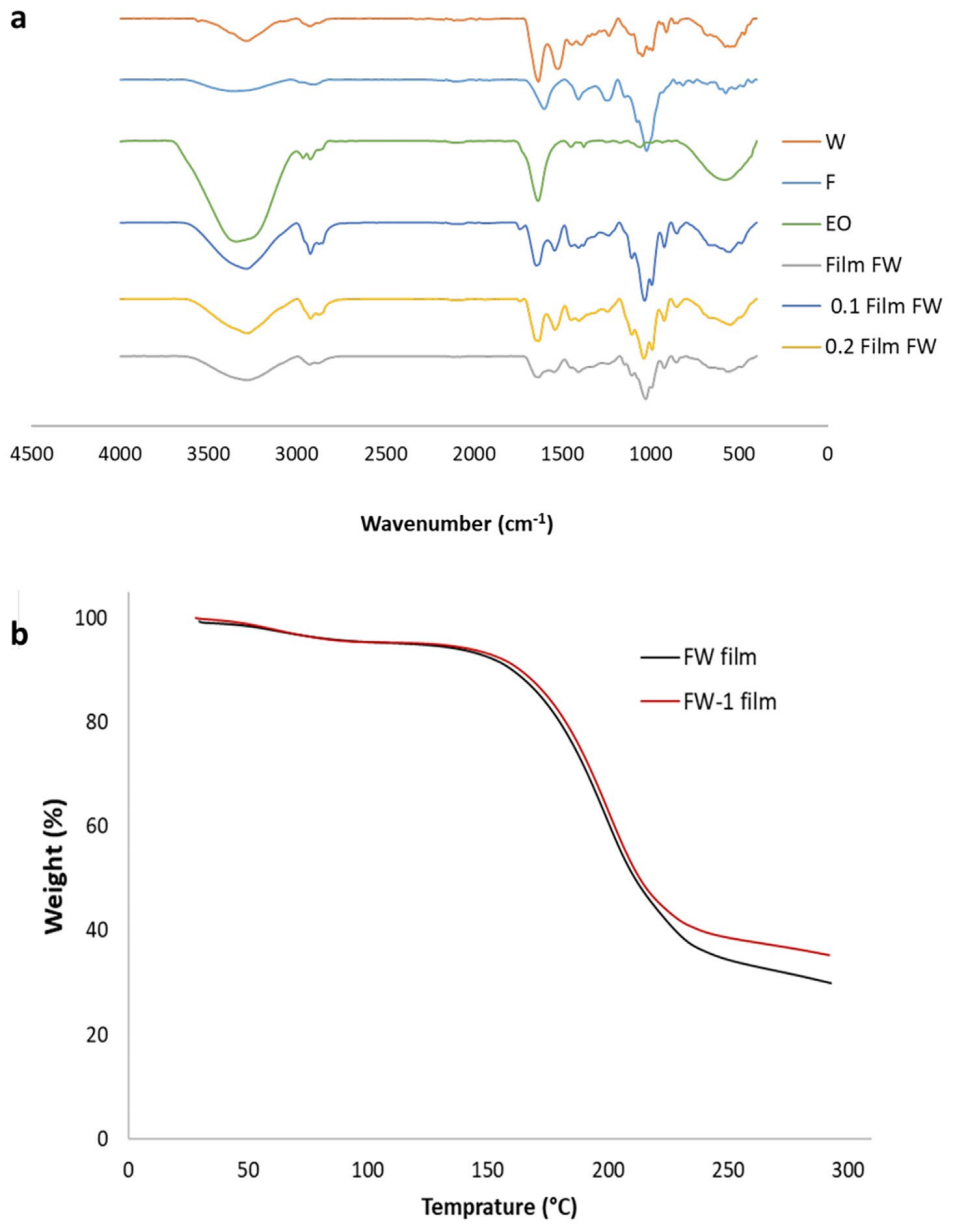
FTIR of fucoidan (F), whey protein (W), geranium EO (EO), and fucoidan‐whey protein films (a); TGA curve of FW film and FW film loaded with 1% geranium essential oil (FW‐1 film) (b).

Figure 2a shows the FTIR pattern of whey protein, fucoidan, and neat and active FW films. FTIR spectrum of whey protein displays characteristic peaks for its proteinaceous nature, mainly due to vibrations from functional and amide groups. The broad peak in the region around 3100–3500 cm^−1^ accounts for O–H and N–H stretching due to hydrogen bonding in the backbone structure of the protein. The Amide I band at 1662 cm^−1^, due to the stretching of the peptide bond's C=O, provides information about proteins' secondary structure (α‐helix, β‐sheet). The Amide II band around 1510 cm^−1^, due to N–H bending and C–N stretching, assists in the characterization of proteins. Peaks between 1050 cm^−1^ account for the C‐O stretching of carbohydrates in whey, present as lactose and glycoproteins. Weak peaks around 2800–3000 cm^−1^ result from the aliphatic side chain's C‐H stretching. These characteristic peaks provide whey protein's structural integrity, peptide bonds, and content of secondary structure.

FTIR spectrum of fucoidan, a sulfated polysaccharide composed mainly of fucose, displays typical peaks due to functional groups. A broad O–H stretch in the region around 3131–3600 cm^−1^ due to hydroxyl (‐OH) groups due to polysaccharide chains. The aliphatic fucose residue's C‐H stretching can be viewed between 2818 and 3000 cm^−1^. 1597 cm^−1^ peak due to asymmetric stretching of carboxylate (‐COO^−^) groups strong peak at 1231 cm^−1^ typical for S=O stretching due to sulfate (‐SO_3_^−^) groups, a typical feature for sulfated polysaccharides. Peaks between 930 and 1141 cm^−1^ comprise various peaks due to stretching of glycosidic bonds' C‐O‐C and sulfate (‐SO_3_^−^) vibrations, with 930–950 cm^−1^ in particular indicating C‐O‐S stretching, confirming sulfate substitution in sugar residues. FTIR spectrum of Geranium EO displays typical peaks due to its chief bioactive compounds such as citronellol, geraniol, and linalool, being monoterpenes and alcohols. The broad O–H stretch around 3200–3600 cm^−1^ identifies hydroxyl (‐OH) groups from alcohols. Stretching vibrations from alkanes and alkenes' C‐H fall in the region 2800–3100 cm^−1^ with peaks around 3000 cm^−1^ for unsaturated C‐H in alkenes. The sharp peak at 1628 cm^−1^ is typically due to alkenes' C=C stretching vibrations, confirming the presence of compounds like geraniol and citronellol with unsaturated structures. The broad peak at 570 cm^−1^ could be due to alkene (C=C) out‐of‐plane bending vibrations or skeletal vibrations from some terpenoid structure. FTIR spectra of a fucoidan‐whey protein film with glycerol contain typical peaks from each of the three components, confirming interactions between them. A broad O–H stretch around 3200–3500 cm^−1^ confirms strong hydroxyl group hydrogen bonding from fucoidan, whey protein, and glycerol. Amide I around 1650 cm^−1^ due to the peptide bond's C=O stretching and amide II around 1550 cm^−1^ due to N–H bending and C–N stretching confirm a protein structure. Peaks around 1220–1260 cm^−1^ due to S=O stretching and around 800–850 cm^−1^ due to S–O bending confirm the presence of sulfate (‐SO_3_^−^) groups from fucoidan. The vibrations from C–O stretching around 1021 cm^−1^ correspond to fucoidan's glycosidic bonds (C–O–C) and to glycerol's C–O stretching, and C–H stretching around 2800–2950 cm^−1^ comes from whey protein and glycerol. The sharp peak around 1634 cm^−1^ could be due to fucoidan‐protein interactions influencing C=C or C=O stretching. These spectral features confirm the formation of a blended film in which fucoidan, whey protein, and glycerol interact via hydrogen bonding and electrostatic interactions, affecting the structural and functional properties of the film.

FTIR spectra for a fucoidan‐whey protein film with glycerol and geraniol EO (0.5% and 1%) exhibit typical peaks from each component with different intensities in proportion to geraniol concentration. A broad O–H stretch around 3100–3500 cm^−1^ results from hydrogen bonding by hydroxyl groups in fucoidan, whey protein, glycerol, and geraniol with a more intense peak in geraniol presence. The amide I band (~1635 cm^−1^) and amide II band (~1540 cm^−1^) indicate peptide bonds and N–H bending in whey protein, and peaks around 1237 cm^−1^ (S=O stretching). The C–O stretching vibrations at 1000–1150 cm^−1^ correspond to glycosidic bonds in fucoidan and glycerol, and to C–H stretching at 2800–2950 cm^−1^ by aliphatic groups in glycerol, whey protein, and geraniol. Peaks due to geraniol result in a C=C stretch around 1635 cm^−1^ and O–H stretch around 3300 cm^−1^ with intensities increasing at 1% geraniol compared to 0.5%. The region below 1500 cm^−1^ in the fingerprint region shows complex peaks influenced by interactions between fucoidan, whey protein, glycerol, and geraniol. Increased concentration of geraniol results in more intense peaks in C=C and O–H stretching, indicating increased interactions and possible modifications in hydrogen bonding in the film.

#### 3.3.6 TGA

TGA thermograms indicating the thermal degradation behavior of FW films in the presence EO nanoemulsion are depicted in Figure 2b. TGA curves were composed of three main stages. The first slight weight loss was observed under 100°C, which was related to the vaporization of free water adsorbed in the films (Ding et al. 2023). A remarkable weight loss was observed at approximately 150°C–220°C (second stage), with maximum degradation rates at 250°C, primarily associated with the loss of glycerol and the decomposition of film compounds (Bhatia et al. 2023). As can be seen, active FW film incorporated with 1% (v/v) EO nanoemulsion had a TGA curve similar to FW film. The weight loss of FW film (control) was 69.409% (3.496 mg), while its thermal stability was slightly improved after the incorporation of EO nanoemulsion. According to the results, FW‐1 had a 64.690% (3.281 mg) weight loss at the final degradation. Our results were in agreement with Ganeson et al. (2022) who observed the higher thermal stability of gelatin activated with emulsion of cinnamon oil. Bhatia et al. (2024) reported that adding grapefruit EO to k‐carrageenan film enhanced thermal degradation, possibly due to the cross‐linking properties of the active polyphenolic ingredients in EO, and created a compact, crystalline structure.

#### 3.3.7 SEM

**FIGURE 3:**
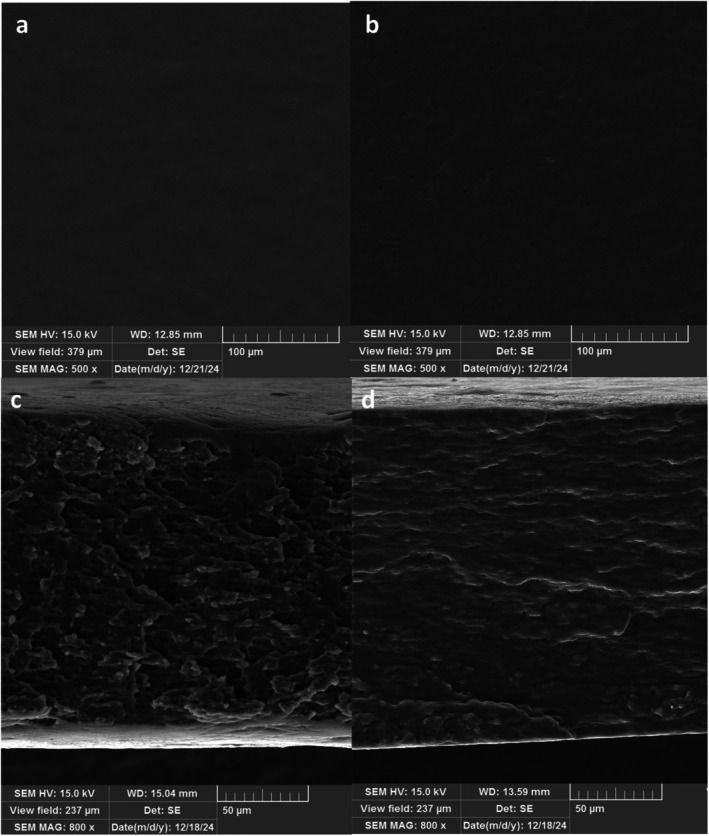
SEM images of fucoidan‐whey protein film (FW film) (a), fucoidan‐whey protein loaded with 1% geranium EO nanoemulsion (FW‐1) (b), and cross‐section of FW film (c) and FW‐1 (d).

The surface and cross‐section images of the FW based films are depicted in Figure 3. The SEM images of both control and active FW film containing 1% geranium EO nanoemulsion showed that all films had very smooth and continuous surfaces. These results indicated that the EO was well distributed and encapsulated in the complex of fucoidan and whey protein film solution, thereby preventing EO evaporation and avoiding changes in film morphology. These results indicated that the EO nanoemulsion was more compatible with fucoidan and whey protein biopolymers. Furthermore, the smooth surface of active films indicated that the nano‐size of emulsion droplets can improve the emulsion stability and protect the aggregation of geranium EO and phase separation during the film drying (Fattahi et al. 2020).

Additionally, the cross‐section of the control FW film (without geranium EO) exhibited a rough, compact structure with some pores. In contrast, the incorporation of EO into the FW films created a relatively denser structure with filled pores and no phase separation, indicating the embedding of EO into the FW film matrix. The higher compactness and denser structure of films were attributed to the presence of Tween 80, which created new interactions with the whey protein and film solution, resulting in higher integrity of the films (Ghadetaj et al. 2018). A similar observation was reported by Amjadi et al. (Ramos et al. 2012), who noted cracks and pores in the cross‐section of the whey protein film after incorporating orange peel ( *Citrus sinensis* ) EO nanoemulsion.

#### 3.3.8 Antioxidant Activity

Table 3 shows the antioxidant activity of neat and active FW films. According to the results, the addition of geranium EO nanoemulsion had a significant effect on the antioxidant ability of FW films (*p* < 0.05). The film without EO nanoemulsion (control) showed moderate antioxidant activity with a DPPH scavenging ability of 35.39% ± 3.19% at a concentration level of 0.02 g of film. The antioxidant activity was considerably enhanced by using geranium EO nanoemulsion in the films. With a concentration increase in geranium EO nanoemulsion, the DPPH scavenging rate was increased to a value of 62.89% when 0.05% geranium EO nanoemulsion was inserted, suggesting a concentration‐dependent activity. A considerably high scavenging ability of 72.91% ± 2.53% was observed in FW‐1 (containing 1% geranium EO nanoemulsion), justifying the highest antioxidant activity of the developed active FW edible film (Table 3).

It is worth noting that synergistic antioxidant activities of fucoidan and geranium EO could be observed. The antioxidant activity of the neat FW film might be attributed to fucoidan. The antioxidant potential of fucoidan is mainly related to its structure, phenol, and sulfate level (Alboofetileh et al. 2019). The antioxidant activity of fucoidan from different seaweeds such as fucoidan derived from *Sargassum hystrix* (Husni et al. 2022), and *Sargassum echinocarpum* (Laeliocattleya et al. 2025) has been confirmed. The antioxidant and radical scavenging ability of fucoidan is strongly attributed to the level of fucose (due to their methyl active groups), and uronic acids (Hifney et al. 2016). In similar, Samani et al. (2023) reported that the incorporation of 0.5% fucoidan increased antioxidant activity of chitosan‐agar films. Moreover, some studies confirmed the antioxidant potential of geranium EO. For instance, Rezig et al. (2024) confirmed the antioxidant activity of geranium EO due to the high content of citronellol and geraniol. Similarly, Ebrahimi et al. (Hifney et al. 2016) also revealed that *Pelargonium graveolens* flower extract had a stronger DPPH radical scavenging ability than the leaf extract, which is attributed to a high content of phenolic compounds.

#### 3.3.9 Antimicrobial Activities

**FIGURE 4:**
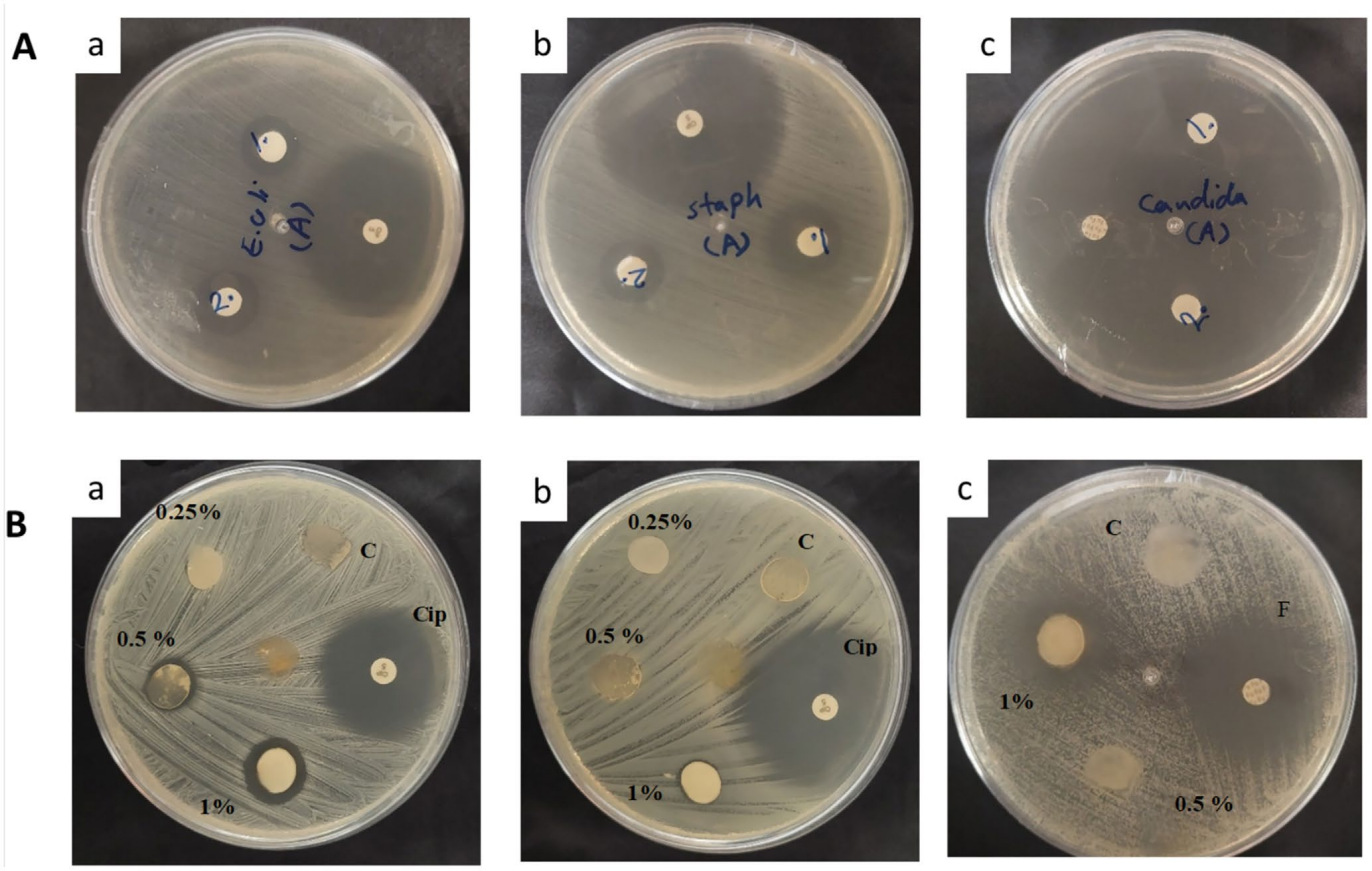
Antimicrobial activity of (A) EO using blank disks loaded with 10 and 20 μL of EO against (a) *E. coli* ; (b) *S. aureus* ; (c) *C. albicans* . Ciprofloxacin and fluconazole disks were tested as controls and (B) antimicrobial activity of fucoidan‐whey protein film containing geranium EO against (a) *E. coli* ; (b) *S. aureus* ; (c) *C. albicans* . Film disks without incorporation of EO and antimicrobial disks (ciprofloxacin: Cip; fluconazole: F) were tested under similar conditions as control.

The inhibitory effects of EO and FW films were evaluated using the agar diffusion method, with antimicrobial potency quantified by clear zone formation around film discs. Figure 4 depicted the antimicrobial activity of neat and active FW films. The results demonstrated potent antimicrobial activity of EO against all tested bacterial and fungal strains. The EO demonstrated significant antifungal activity against *C. albicans* . Also, FW‐1 films exhibited growth inhibition against both Gram‐negative ( *E. coli* ) and Gram‐positive ( *S. aureus* ) pathogens. Moreover, FW‐0.5 and FW‐1 films displayed the significant inhibitory growth on *C. albicans*.

The antimicrobial property of geranium EO is mainly related to its phenolic compounds and its inhibition against *Staphylococcus epidermidis* and two strains of *Saccharomyces cerevisiae* is reported (Wang et al. 2022). However, the differences in cell wall structures between Gram‐positive and Gram‐negative bacteria likely result in varying susceptibilities to films containing EO with antimicrobial activity. Gram‐negative bacteria possess an additional outer membrane containing lipopolysaccharides, which can be exploited by specific compounds to enhance antimicrobial effects (Tavares et al. 2020). Moreover, the antibacterial property of EOs is attributed to their constituents, which enable them to insert into the phospholipid bilayer of the cell wall. It has been reported that the antimicrobial activity of *P. graveolens* EO is mainly attributed to geraniol and citronellol (Wang et al. 2022). Dos Santos et al. (2024) also observed the strong antimicrobial potential of geranium EO against *Listeria monocytogenes* , *S. aureus* , and *E. coli* . The antifungal activity against Aspergilus niger was also claimed by Allizond et al. (2023). Ebrahimi et al. (2025) also prepared an active nano‐coating incorporating leaf and flower extracts of *Pelargonium graveolens* , using fenugreek seed gum and soy protein isolate. Authors reported the antimicrobial potential of the extract against *S. aureus* and *E. coli* and claimed that a coating incorporating the encapsulated extract can retard microbial growth in mutton meat during cold storage. Our results were close to Rezig et al. (2024) revealing the strong antimicrobial activity of geranium EO against *E. coli* and *S. aureus* in comparison with *Serratia marcescens* , *Pseudomonas aeruginosa* and *Enterococcus hirae* . Similarly, Wang et al. (Sayadi et al. 2024) confirmed the antibacterial activity of cassava starch film loaded with geranium EO against *E. coli* , *L. monocytogenes* , and *S. aureus* . Authors claimed that starch film acts as a carrier to slow the release of EO and exhibits prolonged antimicrobial potential.

The moderate antimicrobial activity of geranium EO nanoemulsion in our study was attributed to its encapsulation with nanoemulsion. Because geranium EO should be released from the nanoemulsion first and then transferred to the surface of the film and exhibit an inhibitory effect against bacteria (Abedi et al. 2024). Additionally, nanodroplets of emulsion provide higher surface area and bioavailability of EO to alter the microorganism cell membrane (Cheng et al. 2024). Moreover, the addition of geranium EO into fucoidan film showed synergistic antimicrobial activity, because fucoidan has an inhibitory effect against different bacteria such as *E. coli* , *L. monocytogenes* , and *Salmonella enterica* serovar Typhimurium, depending on the fucoidan content and exposure time (Ayrapetyan et al. 2021; Poveda‐Castillo et al. 2018). The antibacterial property of fucoidan is related to its potential to bind with specific receptors and interfere with cell membrane permeability, resulting in cell leakage and cell death (Poveda‐Castillo et al. 2018). According to our results, films containing 1% geranium EO nanoemulsion have shown significant antifungal activity, which could be attributed to their chemical compositions, primarily citronellol and geraniol. These compounds can disrupt fungal cell membranes and inhibit growth (Nazzaro et al. 2017; Serra et al. 2018; Dzamic et al. 2014). In addition, the concentration of EO in films significantly affects their antimicrobial activity, as mentioned in previous studies (Simsek et al. 2020). Generally, increasing the concentration of EOs in films enhances their antimicrobial properties.

## 4 Conclusion

In the present study, FW edible films loaded with geranium EO nanoemulsion were successfully prepared. The active FW film containing geranium EO nanoemulsion had lower TS and EB values. While, the addition of EO nanoemulsion had no significant effect on the surface of the films and water barrier properties. Additionally, the high antioxidant potential and antimicrobial activities of films were confirmed, and active FW films exhibited strong antioxidant, moderate antibacterial, and antifungal activities dose‐dependently. These results were due to the presence of citronellol, geraniol, and linalool in geranium EO that GC detected. Therefore, active FW edible film incorporated with geranium EO nanoemulsion is a candidate to be used as biodegradable packaging for the shelf life extension of perishable food products. Moreover, there is rare information about fucoidan‐based films and nanofibers. Considering the antibacterial, antifungal and antioxdinat activity of fucoidan, it has potential to be used in combination with other biopolymers as active edible coating/film or active electrospun nanofibers. Moreover, the using of synergistic effect of mixture of EOs to enhance the antioxidant and antimicrobial activities of FW films should be considred. Additionally, the streategies for improving the mechanical attributes of fucoidan films should be developed and using various additives such as clays and crosslinking method are suggested. Therefore, it is necessary to focus on development of novel films or even therapeutic biofilms. Additionally, fucoidan can act as carrier for encapsulation of active agents, thus the development of edible films loaded with fucoidan nanoparticle should be investigated.

## Author Contributions

**Frahang Hameed Awlqadr:** writing – review and editing, methodology. **Kamyar Zomorodian:** writing – review and editing. **Somayeh Yazdanpanah:** writing – review and editing, formal analysis. **Najmeh Oliyaei:** conceptualization, investigation, writing – original draft, methodology, data curation, formal analysis, software, validation. **Aida Iraji:** writing – review and editing, validation, funding acquisition, project administration, resources, supervision. **Cambyz Irajie:** writing – review and editing, formal analysis.

## Funding

The authors would like to acknowledge the support of the Vice‐Chancellor of Research of Shiraz University of Medical Sciences (Grant Number = IR.SUMS.REC.1403.518).

## Conflicts of Interest

The authors declare no conflicts of interest.

## Data Availability

Data will be made available on request.
